# Cervical spine epidural abscess caused by brucellosis: A case report and literature review

**DOI:** 10.1002/ccr3.5644

**Published:** 2022-03-22

**Authors:** Misagh Shafizad, Saeid Ehteshami, Hamidreza Shojaei, Reza Jalili Khoshnoud

**Affiliations:** ^1^ Department of Neurosurgery Imam Khomeini Hospital Mazandaran University of Medical Sciences Sari Mazandaran Iran; ^2^ Department of Neurosurgery Shohadaye Tajrish Hospital Shahid Beheshti University of Medical Sciences Tehran Iran

**Keywords:** brucellosis, corpectomy, epidural abscess, spine

## Abstract

We report a rare case of epidural abscess at the cervical 5‐cervical 6 (C5–C6) levels. The patient underwent surgery with complete abscess removal through C6 vertebral body corpectomy. The result of bacteriological culture was Brucella melitensis. Brucellosis must be considered as a possible cause of epidural abscess in patients from endemic area.

## Graphical abstract

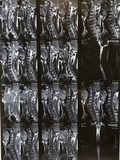

## 1 INTRODUCTION

Brucellosis is a zoonotic bacterial infection caused by Brucella species, which spreads from animals to humans, most often via unpasteurized milk, cheese, and other dairy products.^1^ More rarely, it can be spread through the air or through direct contact with infected animals.^2^ The disease can lead to systemic involvement, and the most common complications are seen in the musculoskeletal system. Musculoskeletal complications are arthritis, bursitis, sacroiliitis, spondylitis, and osteomyelitis. Spinal epidural abscess is a rare complication in the course of spondylitis caused by Brucella species.^3^

Lumbar vertebras are the most common region for epidural abscesses whereas involvement of the cervical spine is uncommon. Management of spinal epidural abscess is controversial.^4^ Several cases of successful treatment with antibiotic alone have been reported in particular in patients with stable neurological condition; however, signs of spinal cord compression represent a neurosurgical emergency because of its potential for causing rapidly progressive paralysis.^5^ In this paper, the authors report a rare case of spinal epidural abscess caused by brucellosis in Iran and then discuss about it.

## 2 CASE PRESENTATION

A 36‐year‐old male patient presented to our neurosurgery department at Imam Khomeini hospital with cervical pain and neck stiffness for about 2 months. At the time, there was a history of fever, fatigue, loss of appetite, malaise, and profuse night sweating for about 10 days. He had been medicated with a few NSAIDs. His medical history was negative for any underlying disease and surgery. He had no history of travel in recent months. On admission, physical examination revealed an initial blood pressure of 130/90 mm Hg, body temperature of 37/8 C, heart rate of 84, and respiratory rate of 16 breaths/minute. Neurological examination revealed motor weakness at the both shoulder abduction, hypoesthesia at the dermatomes of C5‐C6, hyperactive deep tendon reflexes, and no meningeal irritation signs.

**FIGURE 1:**
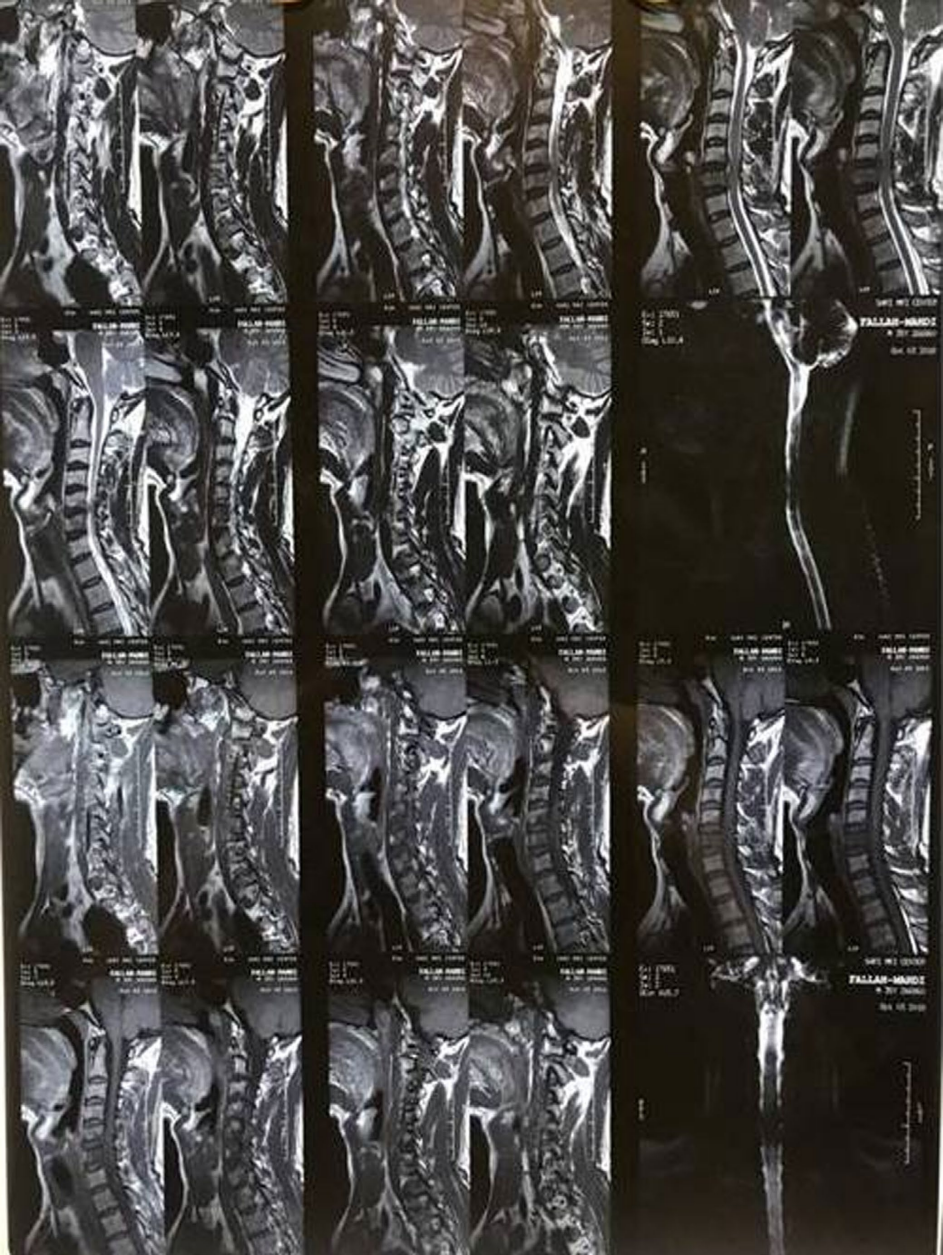
Cervical spine MRI shows an epidural abscess at the C5‐C6 levels

Magnetic resonance imaging of the cervical spine demonstrated spondylitis and epidural abscess formation with a craniocaudal length of 3 cm at the C5‐C6 levels. Decreased spinal canal diameter and minimal spinal cord compression were presented secondary to the epidural abscess (Figure 1). Results of blood tests revealed white blood count (WBC) of 14200, hemoglobin of 13.1 g/dl, erythrocyte sedimentation rate (ESR) of 33, and CRP of 1.3 mg/l. Blood cultures were negative. We tried to rule out tuberculosis and fungal infection; however, the tuberculin skin test became normal and the patient did not appear immune deficient. He was sexually active with his wife of 35 yr. He denied current or previous tobacco use. All relevant immunizations were up to date.

**FIGURE 2:**
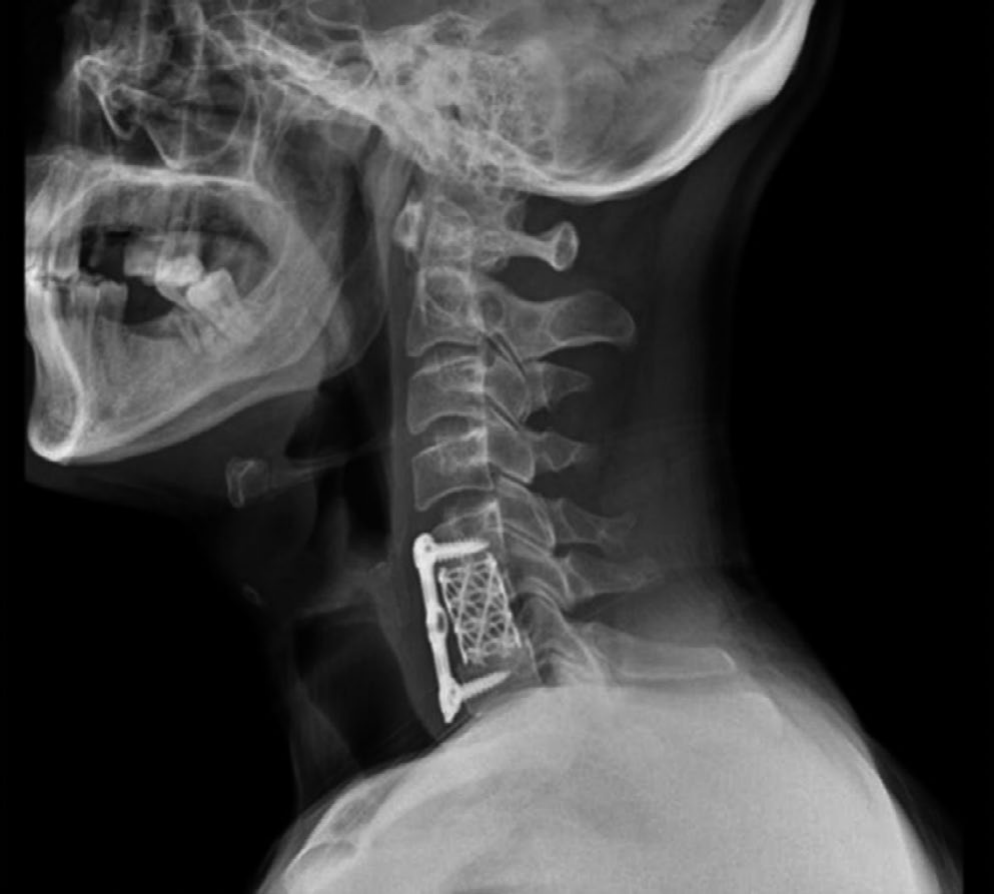
Post‐operative imaging

Because of signs of spinal cord compression, we decide to remove the abscess and perform decompression of spinal cord. The patient underwent anterior cervical surgery with complete abscess removal through C6 vertebral body Corpectomy. Then, reconstruction was done by a corpectomy mesh cage insertion and a cervical plate fixation (Figure 2). The result of bacteriological culture was Brucella melitensis, and pathologic findings were negative for malignancy. After consultation with an infectious disease specialist, effective antibiotic therapy with doxycycline (100mg twice daily) and rifampin (600mg once daily) was considered for 6 months. After surgery, the patient made a good recovery without new neurological deficits and no recurrence. After 6 months follow‐up, the patient had been recovered from all of the previous symptoms.

The present case report conformed to the provisions of the declaration of Helsinki (as revised in 2013). Written informed consent has been obtained from the patient regarding processing personal information and the publication of medical data.

## 3 DISCUSSION

Approximately 500,000 cases of brucellosis are reported annually worldwide, most of which occur in developing countries.^6^ Brucella species is responsible for only 0.1% of cases of spinal epidural abscess. Risk factors include immune‐compromised states such as diabetes mellitus, alcoholism, chronic renal failure, cancer, and acquired immunodeficiency syndrome, as well as spinal procedures including epidural anesthesia or analgesia and spinal surgery or trauma.^7^ No predisposing conditions and specific risk factors for contamination were found in the present case, and also, his familial history was negative for brucellosis and other infections.

Although there were lots of reports about brucellar infection involving the vertebral body or intervertebral disk, there were few previous reports about brucellar infection involving epidural space. Cervical spinal epidural abscess due to brucellosis is a rare condition, difficult to diagnose and can be complicated by disastrous neurological and vascular complications if left untreated.^8^ Hence, early diagnosis and initiation of appropriate medical and surgical treatment are lifesaving.

In the cases of spinal epidural abscess due to Brucella infection, spinal pain with palpation, local tenderness, or fever can be seen clinically. However, these findings are not specific.^9^ In our case, the symptoms were cervical pain, neck stiffness, fever, fatigue, loss of appetite, malaise, and profuse night sweating.

Management of spinal epidural abscess due to brucella spices is not standard and remains controversial. To the best of our knowledge, no case of spinal epidural abscess associated with brucellosis has been reported in Iran earlier. Here, we present a rare case of cervical spinal epidural abscess caused by brucellosis treated with both surgery and antibiotic therapy. Alyousefi et al.^10^ reported first case of cervical epidural abscess caused by brucellosis in Saudi Arabia. Their case presented with fever and back pain and was successfully treated with only antibiotic therapy for 6 months. Boyaci et al.^11^ reported a case of Brucellar spinal epidural abscess in Turkey presented with night sweat and lumbar spondylodiscitis who was treated with antibiotic regimen without surgery.

Song et al.^12^ reported a case of cervical spine epidural abscess causing neurological deficits treated with anterior cervical spine discectomy, fusion with an iliac strut and antimicrobial therapy. In the current case, we used a corpectomy mesh cage for anterior column reconstruction and solid fusion was achieved at follow‐up visit.

Diagnosis of Brucella infection requires isolation of the bacterium from the blood or tissue samples. The optimal antibiotic regimen and duration of treatment for brucellar spinal abscess are still controversial. In general, combination therapy of doxycycline plus streptomycin for at least 12 weeks is accepted more.^13^ Exact follow‐up of patients is important to ensure complete treatment.

## 4 CONCLUSIONS

Brucellar spinal epidural abscess is a rare condition, difficult to diagnose, and can be complicated if left untreated. Brucellosis must be considered as a possible cause of spinal epidural abscess in patients from endemic area. Multidisciplinary management is crucial for the treatment of cervical spine brucellosis.

## CONFLICTS OF INTEREST

The authors declare that they have no conflict of interest.

## AUTHOR CONTRIBUTIONS

M.S designed the study. S.E carried out the literature survey. H.S wrote the manuscript. R.J provided financial support and supervised the work.

## ETHICAL APPROVAL

The present case report conformed to the provisions of the declaration of Helsinki (as revised in 2013).

## CONSENT

Written informed consent was obtained from the patient to publish this report in accordance with the journal's patient consent policy.

## Data Availability

The data that support the findings of this study are available from the corresponding author upon reasonable request.
